# Nutritional Evaluation of Quinoa Genetic Resources Growing in the Climatic Conditions of Central Europe

**DOI:** 10.3390/foods12071440

**Published:** 2023-03-28

**Authors:** Lucie Dostalíková, Petra Hlásná Čepková, Dagmar Janovská, Pavel Svoboda, Michal Jágr, Václav Dvořáček, Iva Viehmannová

**Affiliations:** 1 Department of Crop Sciences and Agroforestry, Faculty of Tropical Agrisciences, Kamýcká 129, 16 500 Prague, Czech Republic; 2 Gene Bank, Crop Research Institute, Drnovská 507/73, 16 106 Prague, Czech Republic; 3 Molecular Genetics, Crop Research Institute, Drnovská 507/73, 16 106 Prague, Czech Republic; 4 Quality and Plant Products, Crop Research Institute, Drnovská 507/73, 16 106 Prague, Czech Republic

**Keywords:** breeding, *Chenopodium*, protein, mass spectrometry, phenolic compounds, quinoa

## Abstract

Quinoa displays huge genetic variability and adaptability to distinct climatic conditions. Quinoa seeds are a good source of nutrients; however, the overall nutritional composition and nutrient content is influenced by numerous factors. This study focused on the nutritional and morphologic evaluation of various quinoa genotypes grown in the Czech Republic. Significant differences between years were observed for morphological traits (plant height, inflorescence length, weight of thousand seeds). The weather conditions in the year 2018 were favorable for all the morphological traits. The protein content of quinoa accessions ranged between 13.44 and 20.01% and it was positively correlated to mauritianin. Total phenolic content varied greatly from year to year, while the antioxidant activity remained relatively stable. The most abundant phenolic compounds were the flavonoids miquelianin, rutin, and isoquercetin. Isoquercetin, quercetin, and N-feruoloyl octopamine showed the highest stability under variable weather conditions in the analyzed years. A total of six compounds were detected and quantified in quinoa for the first time. Most varieties performed well under Central European conditions and can be considered a good source of nutrients and bioactive compounds. These data can be used as a source of information for plant breeders aiming to improve the quality traits of quinoa.

## 1. Introduction

Quinoa (*Chenopodium quinoa* Willd.) is a pseudocereal from the Chenopodiaceae family, with its origin located around Lake Titicaca, lying on the border of Peru and Bolivia. Thanks to its long-term domestication and the various farming activities of ancient societies living in the Andean range [1], quinoa today displays a huge genetic variability. This allows quinoa to adapt to different abiotic stresses [2,3] and opens the possibility of cultivation in relatively distinct climatic conditions worldwide [4].

Thanks to its resilience, quinoa can be sustainably produced in marginal environments, which is a crucial trait, because salinization and aridity are predicted to increase in most parts of the world. It is estimated that climate change will negatively impact food safety in low-income countries relying primarily on agriculture and with limited inputs. Therefore, quinoa might be, together with other indigenous foods, a significant tool in fighting against hunger and malnutrition [5].

Quinoa seeds and leaves are consumed in the form of traditional and novel food products and beverages [6]. Thanks to the presence of valuable nutrients, quinoa can be used for the improvement of the nutritional profile of gluten-free products [7]. Quinoa contains a good amount of minerals and vitamins, together with a relatively high amount of nutritionally valuable oil, with a predominance of health-beneficial polyunsaturated fatty acids [8]. Thanks to its exceptional features and characteristics, quinoa starch has interesting physiochemical properties, allowing its potential use in a broad spectrum of food products. Quinoa is further prized for its relatively high seed protein content, with the presence of all essential amino acids [9,10]. In addition to the primary metabolites, quinoa contains numerous secondary metabolites, divided into five groups: phenolic acids, flavonoids, terpenoids, steroids, and nitrogen-containing metabolites. The majority of them are biologically active, possessing, for example, anticancer [11,12], immunoregulative [13,14], antimicrobial [15], and anti-inflammatory properties [16,17]

On the other hand, the reported nutritional composition and nutrient content of quinoa is highly variable throughout the literature. Besides the effect of genotype, nutrient content and composition of quinoa were previously reported to be influenced by agroecological conditions [18,19,20], as well as the metabolomic and morphological characteristics of the plant itself [21].

It is necessary to broaden the current knowledge of quinoa, by analyzing and evaluating the wide range of quinoa genetic resources, which will serve as a great source of information about which quinoa genotypes have the potential to be cultivated intensively and which should be improved. This study evaluated an extensive collection of 41 quinoa genotypes grown for 4 consecutive years (2018–2021) under the climatic conditions of the Czech Republic. The main aim was to characterize the chemical and nutritional compositions, together with the agro-morphological traits, of selected varieties with the best performance under Central European climatic conditions. The data obtained will provide necessary and detailed information for further quinoa breeding purposes.

## 2. Materials and Methods

### 2.1. Plant Material

A total of 41 quinoa accessions were subjected to analysis. All the accessions were provided from the U.S. National Plant Germplasm System operated by USDA. During consecutive years 2018–2021, the genotypes were sown on the experimental fields of the Crop Research Institute, v.v.i., in Prague—Ruzyně, Czech Republic. All accessions were sown in two rows 1 m in length, 25 cm apart, and with 50 seeds per row. No pesticide or fungal control was applied. The morphological characteristics of the plants were evaluated according to the descriptors for quinoa and wild relatives. The plant height and inflorescence length measurements were performed in 5 randomly selected plants in each genotype. Seeds were harvested at full maturity. The seeds were dried, cleaned, and stored for further analysis.

### 2.2. Weather Conditions

**Figure 1:**
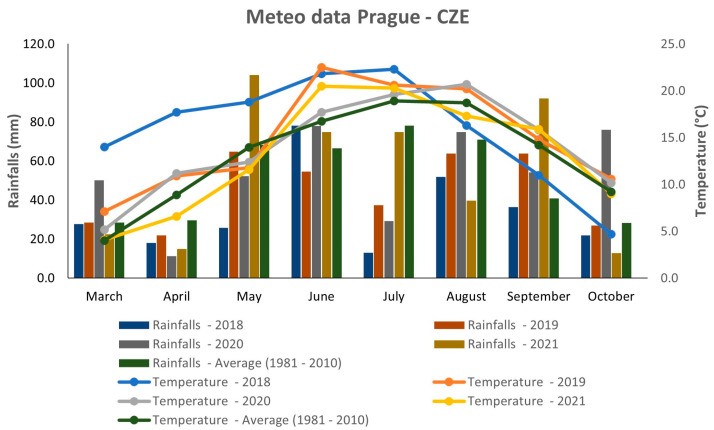
Weather conditions in 2018–2021 in Prague, Czech Republic.

Figure 1 describes the weather conditions during four consecutive years 2018–2021. The meteorological data were gathered from the agrometeorological station at Crop Research Institute, Prague—Ruzyně, Czech Republic. In general, there were variable weather conditions during the analyzed years (Figure 1). The year 2018 showed extremely hot weather during the first half of the growing season; however, in the second half, the mean temperature was the lowest compared to all analyzed years and the 30-year average. This year was also the driest, because the precipitation rate was lower than the 30-year average (1981–2010) during all months, except for June. Extremely dry conditions were observed during May and July 2018. The years 2019 and 2020 had relatively similar temperature patterns, except for June, when the temperature was significantly higher in 2019. In terms of rainfall, the average precipitation rate was quite variable in both years. Relatively abundant rainfall occurred in June, August, and October 2020, whereas May and July were drier, with precipitation rates lower than the 30-year average. In 2019, there was relatively high precipitation during September, but the other months reached values that were comparable to or lower than the 30-year average. Overall, the year 2019 can be considered the warmest of all studied years and drier compared to 2020. In terms of mean temperature, the year 2021 was more or less comparable to what was seen in 2019 and 2020, except for June and August. In contrast, the precipitation rate showed several extremes in 2021. The most abundant rainfall occurred during May and September, whereas a low amount of rainfall was observed in August and October. The precipitation rate of the two resting months (June and July) was comparable to the 30-year average.

### 2.3. Chemicals

Standards of the phenolic compounds 2-OH cinnamic acid, 4-OH benzaldehyde, apigenin, caffeic acid, catechin, chlorogenic acid, emodin, epicatechin, gallic aicd, genistein, glycitein, hesperidin, homoorientin, isoquercetin, isovitexin, isorhamnetin, kaempferol, luteolin, n-feruloyl octopamine, naringenin, neochlorogenic acid, mauritianin, miquelianin, orientin, *p*-coumaric acid, pinocembrin, quercetin, quercitrin, rhamnetin, rutin, salicylic acid, taxifolin, umbelliferone, vitexin, and the internal standard probenecid and verapamil hydrochloride were purchased from Sigma–Aldrich (St. Louis, MO, USA). Methanol (LC-MS grade, ≥99.9%) was obtained from Riedel de Haën (Seelze, Germany). Formic acid (LC-MS grade, 99%) was purchased from VWR (Leuven, Belgium). Pure water was attained from a Milli-Q purification system (Millipore, Bedford, MA, USA).

### 2.4. Sample and Standard Preparation

To prepare reference stock solutions, reference standards of each phenolic compound were dissolved in methanol, to obtain stock solutions of 0.5 mg/mL. The reference stock solutions were stored at −18 °C. The calibration curves for the phenolic compound quantification were prepared by dilution of stocks, with a methanol concentration range of 0.001–2.000 µg/mL. Furthermore, probenecid and verapamil were dissolved in methanol at 0.5 mg/mL, to prepare a stock solution of the internal standard. Internal standards were then added to the individual reference standard solutions or test samples, to a final concentration of 0.1 µg/mL.

The seeds of quinoa were milled with an IKA A11 basic mill (IKA-Werke, Staufen, Germany), and the flour was stored in a dark cold place (4 °C) in well-sealed plastic bags.

For the mass spectrometric analysis, the extraction of seed samples was based on the method described by Janovská et al. [22]. Briefly, 0.1 g of the whole meal flour was extracted twice with 1 mL of extraction solvent (80% methanol with probenecid and verapamil as internal standards at a concentration of 0.1 µg/mL) in Eppendorf tubes for 60 min at 45 °C and using an ultrasonic bath. Samples were then centrifuged for 10 min at 13,500 rpm. Obtained supernatants from each sample were filtered through 0.2 µm nylon syringe filters (Thermo Scientific, Rockwood, TN, USA). Extracts were prepared a maximum of 2 days before the UHPLC-ESI-MS/MS analysis and stored at −18 °C.

### 2.5. UHPLC-ESI-MS/MS Instrumentation

The chromatographic system (Dionex UltiMate 3000 UHPLC system, Dionex Softron GmbH, Germering, Germany) consisted of a binary pump (HPG-3400RS), an autosampler (WPS-3000RS), a degasser (SRD-3400), and a column oven (TCC-3000RS). Detection was carried out on a quadrupole/orbital ion trap Q Exactive mass spectrometer (Thermo Fisher Scientific, San Jose, CA, USA). Analytes were separated on a reversed-phase Ascentis Express C18 column (2.1 mm × 100 mm, 2.7 µm) from Supelco (Bellefonte, PA, USA). The LC-MS system was equipped with a heated electrospray ionization source (HESI-II) and Xcalibur software, version 4.0 (Thermo Fisher Scientific, San Jose, CA, USA).

### 2.6. UHPLC-ESI-MS/MS Analysis

Chromatographic separation was carried out using gradient elution, with 0.2% formic acid (*v*/*v*) in water as solvent A and methanol with 0.2% formic acid (*v*/*v*) as solvent B. The LC gradient started with 99% of solvent A + 1% of solvent B; followed by gradient elution to 40% A + 60% B at 11 min. The column was eluted with 100% of solvent B for 2 min. Equilibration was achieved by washing the column with 99% A + 1% B for 2 min. The total analysis took 15 min. The column was maintained at 40 °C at a flow rate of 0.35 mL/min. The injection volume was 1 µL.

The mass spectrometer analysis was run in negative ESI mode. The spray voltage was maintained at −2.5 kV. The sheath gas flow rate was 49 arbitrary units, the auxiliary gas flow rate was 12 arbitrary units, and the sweep gas flow rate was 2 arbitrary units. The capillary temperature was 260 °C. Nitrogen was used as the sheath, auxiliary, and sweep gas. The heater temperature was maintained at 419 °C. The S-lens RF level was 30. The precursor ions in the inclusion list were isolated within the retention time window of ±60 s, filtered in the quadrupole at the isolation window (target *m*/*z* ± 0.8 *m*/*z*), and fragmented in an HCD collision cell C-trap at a resolution of 17,500 FWHM (full width at half maximum) resolution, an AGC target value of 1 × 106, and a maximum injection time of 50 ms. The normalized collision energy (NCE) was optimized for each compound. The precursor and daughter ions monitored, retention times, and NCE values are shown in Table S1. The precision and calibration of the Q Exactive Orbitrap LC/MS/MS instrument were examined using a reference standard mixture obtained from Thermo Fisher Scientific. The measurements were performed in three replicates. Data were evaluated with Quan/Qual Browser Xcalibur software, version 4.0.

### 2.7. Determination of the Phenolic Compound Concentration in Quinoa Samples

Identification of phenolic compounds in quinoa samples was based on their retention times relative to the authentic standards and mass spectral data (accurate mass determination generating elemental composition and fragmentation patterns of a molecular ion) obtained through LC-MS/MS, most were compared with those described in our previous studies [22]. Calibration curves were constructed by plotting the peak area (adjusted with probenecid and verapamil as internal standards) versus the concentration of the relevant reference standards.

### 2.8. Chemical Analyses

The dry matter (DM) content of seed samples (5 g) was further dried in an electric hot-air drier at 105 °C for 4 h, according to the standard method [23]. The content of crude protein from each sample was determined using the classic Kjeldahl mineralization method and calculated with a conversion factor of 6.25 [24]. The protein content measurements were performed in two replicates. The results were expressed as % in DM. Total phenolic content (TPC) was determined using Folin–Ciocalteau reagent according to Holašová et al. [25] with slight modifications. The results of the TPC analysis were expressed in grams gallic acid equivalent (GAE) per kilogram of sample dry weight (DW) (GAE g/kg dw). The antioxidant activity (AA) of the samples was determined using a DPPH assay [26]. The results of the DPPH assay were expressed in millimoles of Trolox equivalent (TE) per gram of sample dry weight (DW) (μmol TE/g DW).

### 2.9. Statistical Analyses

Selected morphological descriptors for the whole collection of 41 genotypes were measured in 3 biological replicates. Statistical analysis was performed in the R program (R Development Core Team 2020) and Microsoft Office Excel v. 2016. A two-way analysis of variance (ANOVA) was applied to the data, to test whether there was a significant effect of year and genotypes on the evaluated traits. To compare each accession concerning each descriptor, the means and the standard deviations for each descriptor were calculated separately for each accession and year of observation. Boxplots were also generated, to compare the distribution of values for a set of 22 descriptors between individual years of observation. Years with significantly different means were determined with a Tukey HSD test. Spearman’s rank correlation was also calculated for each pair of descriptors based on the mean values. The correlation test function was applied to test whether the correlation coefficient was significantly different from zero. Furthermore, a heatmap was created for selected traits using the Complex Heatmap package, to display differences between genotypes. Each genotype was color-coded from max (red) to min (blue) based on the values of the respective descriptors in individual years, and a boxplot showing the distribution of values across individual years and genotypes was plot. Heatmaps were combined with a dendrogram based on the average linkage clustering of the Euclidean distance dissimilarity matrix of the values for the respective traits. Summarized data of the evaluated traits and nutritive compounds (means and standard deviation) for the tested genotypes in all years are presented in Table S2. To show the association among samples, data for a set of 19 descriptors were used for the principal component analysis (PCA). Prior to the PCA, the data were scaled, and missing values imputed using the missMDA package. The quality of representation of the variables on the factor map was also calculated for the first two components with the largest variance.

## 3. Results and Discussion

### 3.1. Weather Conditions

The weather conditions during the four consecutive years 2018–2021 showed several extremes in temperature and precipitation, mostly during the years 2018 and 2021 (Figure 1). The years 2019 and 2020 had relatively similar characteristics; however, they both were different from the years 2018 and 2021. The effect of the environment on plant morphology and seed quality is undebatable. As described previously, the growing conditions during the year can significantly affect important quinoa traits, such as the yield [27], fiber content [19], protein and amino acid content [10,28], as well as metabolomic composition [29,30].

### 3.2. Morphological Evaluation

**Figure 2:**
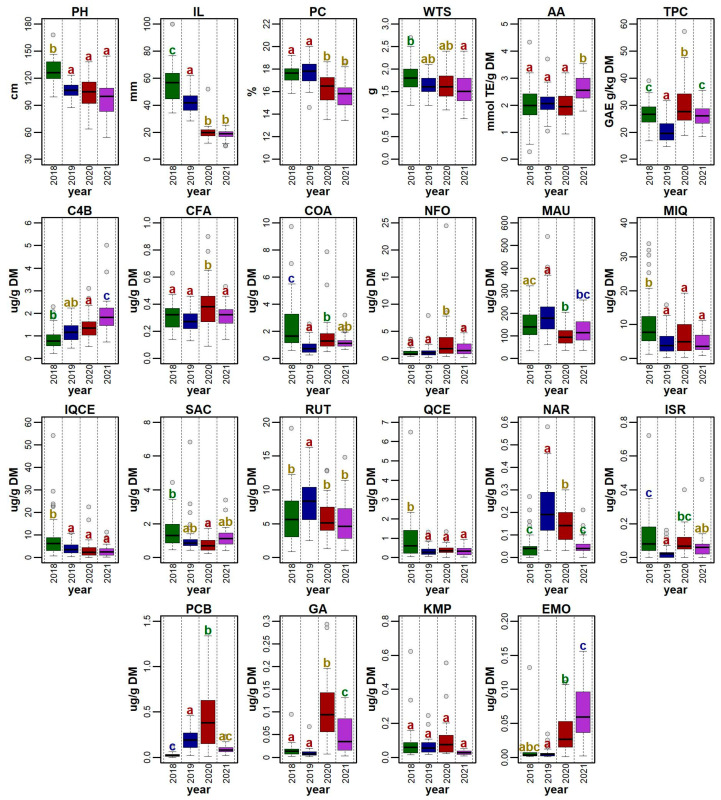
Distribution of values for a set of 22 morpho-phenological parameters and chemical compounds observed for 41 quinoa genotypes grown in the Czech Republic between 2018 and 2021. For each descriptor, the values recorded for each accession in a given year were used for the plot. Boxplots show the distribution of values, with grey shaded points representing outlier values. Significant differences in means between years are denoted by the different letters (Tukey HSD) above each boxplot. The abbreviations for the selected descriptors are as follows: plant height (PH), inflorescence length (IL), protein content (PC), weight of thousand seeds (WTS), antioxidant activity (AA), total polyphenols (TPC), 4-hydroxybenzaldehyde (C4B), caffeic acid (CFA), *p*-coumaric acid (COA), N-feruloyloctopamine (NFO), mauritianin (MAU), miquelianin (MIQ), isoquercetin (IQCE), salicylic acid (SAC), rutin (RUT), quercetin (QCE), naringenin (NAR), isorhamnetin (ISR), pinocembrin (PCB), gallic acid (GA), kaempferol (KMP), and emodin (EMO).

In this study, all genotypes were evaluated under field conditions using the descriptors for quinoa *Chenopodium quinoa* Willd. and wild relatives [31]. The selected descriptors were plant height (PH), inflorescence length (IL), and the weight of thousand seeds (WTS). The mean PH value was the highest in 2018 (127.65 ± 13.77 cm) and the lowest in 2021 (97.88 ± 20.63 cm). A statistically significant difference between the years was only noticed in the year 2018; other years had no significant differences. Statistical differences also existed among genotypes (Figure 2).

**Figure 3:**
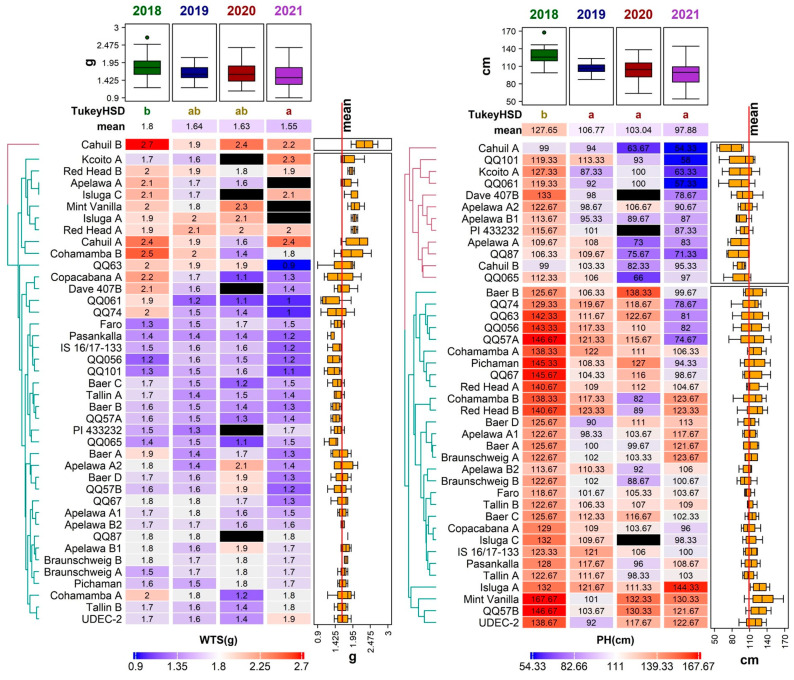
Diversity of 41 quinoa genotypes in terms of weight of thousand seeds (WTS, (**left**)) and plant height (PH, (**right**)) values, illustrated using a heatmap combined with a dendrogram based on average linkage clustering of the Euclidean distance dissimilarity matrix. Values for the respective traits are displayed on a scale from blue (min) to red (max), according to color key below each heatmap. Black rectangles indicate missing values for a given trait in a given genotype. Years with significantly different means are denoted by the different letters (Tukey HSD) above the individual columns of the respective heatmaps. Boxplots above each heatmap show the distribution of values across all accessions in individual years, while the boxplots next to each heatmap show the distribution across all years for individual accessions. The line crossing the side boxplots marks the mean of all values.

The height of a plant is, among other factors, strongly influenced by genotype [32]. This study detected maximum PH in the ‘Mint Vanilla’ (167.67 ± 3.68 cm) in 2018 (Figure 3). This genotype steadily obtained top PH values in almost all studied years, except for 2019. A similar a range of quinoa heights were found in the scientific literature. Thiam et al. [33] reported the range of studied quinoa genotypes at 34.85–127.35 cm, whereas Tabatabei et al. [34] evaluated a broader range (17.20–145.25 cm).

**Figure 4:**
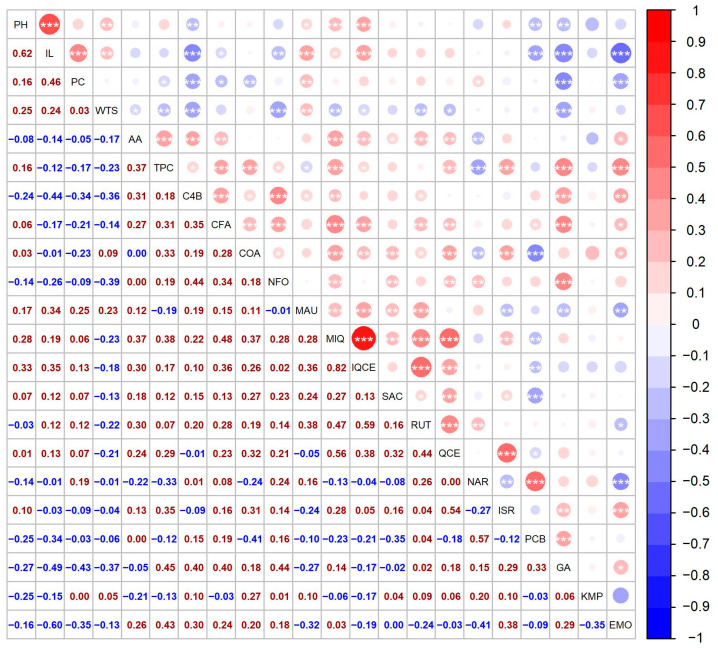
Spearman’s correlation between 22 descriptors for a collection of 41 quinoa genotypes. The circles above the diagonal indicate whether the correlation between the pair of descriptors was negative (red) or positive (blue), while their size represents the magnitude of the correlation, as indicated by the color key and the Spearman’s ρ values below the diagonal. Significant correlations are denoted by * (*p* < 0.05), ** (*p* < 0.01), and *** (*p* < 0.001), respectively.

Relatively high and stable PH values among three studied years (2018, 2019, and 2020) were noticed in genotype ‘QQ57 A’, with the mean PH at 114.58 ± 25.83 cm. A very low variation in PH between years was described in genotypes ‘Tallin B’ and ‘Faro’. The height of the plant was positively correlated to WTS (0.25) (Figure 4). The PH is known to positively correlate to overall seed yield and seed size [35,36].

The heritability of PH reached up to 73%, which makes this trait a point of interest for a further selection of promising lines and yield improvement [37]. However, accessions with a great height (>176.72 cm) and long panicles (>57.94 cm) tend to have lower yields and smaller seed sizes [35]. Excessive plant height may also result in yield losses, caused by lodging [38].

Mean IL was the highest in 2018 (56.11 ± 15.21 cm) and lowest in 2021 (18.51 ± 3.90 cm). The result of the Tukey HSD showed statistical differences between the years, but there was no statistical significance between 2020 and 2021 (Figure 2). The longest inflorescence was recorded in the genotype ‘QQ57 A’ (99.67 ± 40.00 cm) in 2018; however, this genotype did not perform well in any other year. A relatively low variability in this trait was detected in the genotype ‘Dave 407B’. Tabatabaei et al. [34] reported similar values, ranging between 7.05–71.75 cm, in 468 quinoa accessions. Slightly different ranges of inflorescence length were observed in different environments: 36.90–120.70 cm [37] and 29.70–62.70 cm [39].

The year 2021 was not suitable for inflorescence development, since almost 50% of the cultivated genotypes had below-average values of panicle length (less than 18.51 cm). The correlation analysis showed a relatively strong positive association (0.62) with the height of the plant (Figure 4), which is in agreement with other authors [35,39,40].

Regarding all four studied years, the WTS ranged between 0.90 g (genotype ‘QQ63’ in 2021) and 2.74 g (genotype ‘Cahuil B’ in 2018). Significant differences were detected between 2018 and 2019 and between genotypes (Figure 3). Compared to several other experiments conducted in Europe, the WTS values in this study were relatively low. For example, the WTS reported in Poland, Belgium, Germany, Italy, and Spain ranged between 1.20 and 3.68 g [18,41]. The most favorable year for this trait was 2018 (mean WTS 1.80 ± 0.32 g) (Figure 3). On the other hand, most genotypes had relatively low WTS in 2021, except for ‘Cahuil A’, ‘Kcoito A’, ‘PI 433232’, ‘Pichaman’, ‘Tallin B’, and ‘UDEC-2’, which had a higher WTS in this year compared to the other three years.

Genotype ‘Cahuil B’ showed above-average performance in WTS, with values ranging between 1.87 and 2.74 g in all studied years Several genotypes in this paper showed relatively stable WTS values during all years of analysis (‘Red Head A’, and ‘Red Head B’); however, the lowest variability was observed in the genotype ‘QQ87’ achieving approximately 1.80 g among all years of analysis. As previously reported, the WTS contributes to overall quinoa yield [33,41].

The overall genotype performance depends more or less on the genetic makeup, environment, and their interactions [32,33]. A proper understanding of quinoa germplasm and its adaptation to various environments is crucial for effective breeding programs and cultivar development [42].

Relatively high temperatures in the first half of the year 2018 and a below-average precipitation rate during most of the growing season were relatively suitable for the majority of quinoa genotypes, since most of the accessions had their best performance in this year for all analyzed morphological traits. Although quinoa response to dry and hot environments is well documented [19,43,44], the negative effect of high precipitation is not well described. In this study, extreme rainfall in 2021 and relatively high precipitation in 2020 affected the studied morphological traits in the majority of the analyzed genotypes. High rainfall may have caused waterlogging stress during both years and the plants may have suffered from unsuitable soil conditions, combined with nutrient deficiency causing the poor performance of the studied genotypes [45,46]. Increased humidity probably contributed to a higher incidence of fungi diseases, which may also have modified the performance of the quinoa accessions during those years.

### 3.3. Crude Protein Content

**Figure 5:**
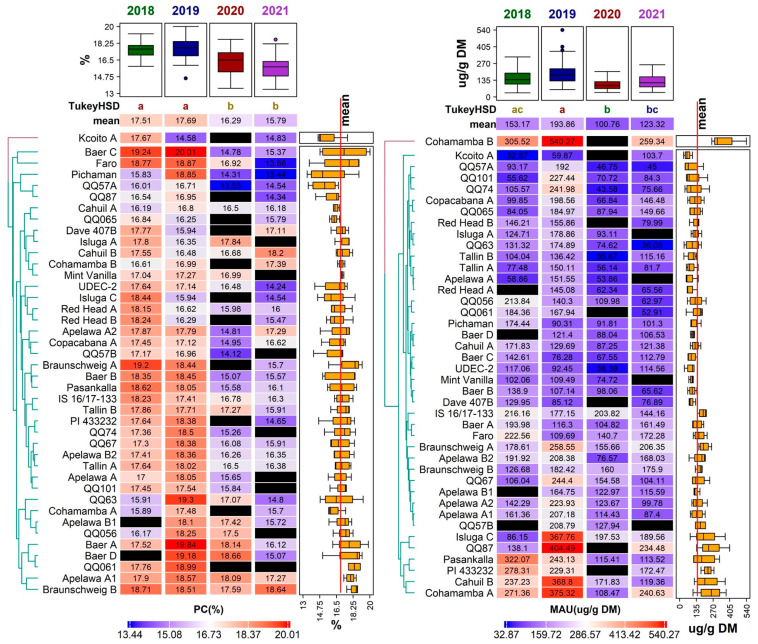
Diversity of the 41 quinoa genotypes in terms of protein content (PC, (**left**)) and mauritianin content (MAU, (**right**)) values, illustrated using a heatmap combined with a dendrogram based on average linkage clustering of Euclidean distance dissimilarity matrix values for the respective traits, displayed on a scale from blue (min) to red (max) according to the color key below each heatmap. Black rectangles indicate missing values for a given trait in a given genotype. Years with significantly different means are denoted by the different letters (Tukey HSD) above the individual columns of the respective heatmaps. Boxplots above each heatmap show the distribution of values across all accessions in individual years, while the boxplots next to each heatmap show the distribution across all years for individual accessions. The quality of representation of the variables is shown in the factor map.

Protein content (PC) fluctuated between 13.44 ± 0.12% in DM (genotype ‘Pichaman’ in 2021) and 20.01 ± 0.17% in DM (genotype ‘Baer C’ in 2019). According to the Tukey HSD results, there were no significant differences between the years 2018 and 2019, and between the years 2020 and 2021 (Figure 5). The values gathered in this study were similar to several other trials on quinoa grown in Europe, such as those reported in Belgium (12.10–18.80% in DM) [18] and Spain (13.20–20.40% in DM) [19,47], but higher than those reported in Poland (12.40–15.98 g/100 g in DM) [48] and Germany (11.90–16.10% in DM) [41].

The highest mean PC was reported for the year 2019 (17.69 ± 1.14%) (Figure 5). On the other hand, the lowest mean PC (15.79 ± 1.19%) was analyzed in 2021. Overall, 56% of genotypes achieved the highest PC in 2019 and almost 37% of genotypes reached the highest PC in 2018. In comparison, only two genotypes (‘Cahuil B’, ‘Cohamamba B’) had the highest PC in 2021 and one genotype in 2020 (‘Isluga A’). Even though some genotypes had low mean PC values, the amount of crude protein was still higher than in most cereals, such as wheat (12%), oat (13%), and rice (7%) [49]. In addition to a balanced [50] or ‘nearly balanced’ amino acid composition [10], quinoa is a great and valuable source of protein for human nutrition.

The observed variation in PC can possibly be explained by environment and/or genotype–environment interactions. The year 2019 was characterized as the warmest of all analyzed years. The precipitation rate in this year was the second lowest of all studied years. Heat stress and slight water stress may enhance the protein content in seeds [19]. Although significant water stress can cause a decrease in the PC [51]. The high precipitation rate during 2020 and 2021 was more harmful in our case. The effect of heavy rainfall and potential waterlogging on protein content is not well documented in quinoa specifically; however, research carried out on winter wheat and red clover concluded that there was a decrease in protein content with high water levels [52,53,54,55].

Despite the influence of the environmental conditions, several genotypes showed relatively low variability in the amount of protein (Figure 3). Very stable results for PC throughout the studied years were observed in the genotypes ‘Mint Vanilla’, ‘Cahuil A’, ‘Cohamamba B’, ‘Braunschweig B’, and ‘Apelawa A1’ (Figure 5).

A medium contribution to the amount of protein was noticed in IL (0.46) (Figure 4). Contrarily, Granado-Rodriguez et al. [47] reported a negative correlation between panicle size and protein content. Furthermore, protein content was positively associated with mauritianin content (0.25). A negative association was observed with emodin (−0.35) and gallic acid (−0.43) (Figure 4).

### 3.4. Total Phenolic Content

The TPC value ranged between 14.74 ± 0.34 GAE mg/g in DM (genotype ‘QQ101’ in 2019) and 57.25 ± 2.87 GAE mg/g in DM (genotype ‘Mint Vanilla’ in 2020). The Tukey HSD showed a significant difference between years; however, the years 2018 and 2021 were not statistically different (Figure 2). The analyzed range of TPC in this investigation was higher than that determined previously. Generally, the TPC fluctuated between approximately 2 and 15 GAE mg/g in the DM in quinoa samples [56,57,58].

The highest mean TPC was recorded in the year 2020 (30.56 ± 9.20 GAE g/kg DM). The majority of genotypes reached the highest TPC this year in comparison to what was measured in the other years (Figure 2). The lowest mean TPC was measured in the year 2019 (20.34 ± 4.06 GAE g/kg DM). The highest stability in TPC values was reported for the genotypes ‘Red Head B’, ‘Apelawa A’, ‘Isluga C’, and ‘PI 433232’. The variety and origin of the sample may significantly affect quinoa metabolomics and final polyphenol content [59]. The observed variations in TPC could have been caused by the reaction of the plant to abiotic stress [60,61]. As suggested by Toubali et al. [29], drought stress decreases the TPC by up to 76%. Nonetheless, this conclusion does not apply to our results, since the driest year was 2018, while the TPC for this year was the second highest.

In this study, several factors contributed to the overall TPC. Correlation analysis showed a weak or medium positive association between TPC and the majority of the metabolites. The strongest contributors to TPC were emodin (0.43) and gallic acid (0.45) (Figure 4). TPC was also positively correlated with AA; however, the association was medium (0.37).

### 3.5. Antioxidant Activity

The highest mean AA (2.59 ± 0.74 μmol TE/g DM) was measured in 2021 (Figure 2) and the lowest mean AA was measured in 2020 (1.95 ± 0.48 μmol TE/g DM). Among all the accessions, the highest AA value was determined for ‘Faro’ (3.54 μmol TE/g DM) in 2021 and the lowest for ‘Cahuil A’ (0.28 μmol TE/g DM) in 2018. The obtained results are difficult to compare with the current scientific literature, since the authors used a different method (e.g., FRAP, ABTS, FIC) and/or expression of the measured values.

In terms of the trait stability, very similar values throughout the years were obtained in genotypes ‘QQ056’, ‘QQ57B’, and ‘Isluga A’; nonetheless, all the genotypes did not reach full maturity in 2021. The number of chemical components related to antioxidant properties varies under different cultivation areas and depends on the genotype–environment interactions [62,63]. In our case, the stress was probably caused by the extreme precipitation rate during 2021 and the higher incidence of fungal diseases.

A weak or moderate positive association was determined between AA and the majority of the analyzed metabolites. The strongest contributor to AA was miquelianin (0.37) and 4-hydroxybenzaldehyde (0.31) (Figure 4).

### 3.6. Composition and Content of Phenolic Compounds

A total of 34 metabolites were evaluated in this study. From this number, a total of 13 compounds were detected in all analyzed genotypes and 15 compounds were detected in trace amounts and/or only in some genotypes. Six compounds were not detected in any of the studied genotypes. To our knowledge, a total of six compounds (2-OH-cinnamic acid, homoorientin, luteolin, naringenin, N-feruloyloctopamine, and 4-OH-benzaldehyde) had never been identified or quantified in quinoa before.

The chemical classes detected in this study were flavones (7 compounds), phenolic acids (7 compounds), flavonols (6 compounds), and flavanols (3 compounds). In addition, groups of hydroxybenzaldehydes, flavans, flavanones, anthraquinones, and methoxybenzaldehydes were detected in quinoa, each represented by one compound.

The results of quantification showed that the most dominant compounds throughout the analyzed years were mauritianin, miquelianin, rutin, and isoquercetin. This was not in agreement with other sources, which considered quercetin and kaempferol as the two major flavonoids in quinoa [64,65,66]. The rest of the analyzed compounds had a mean concentration lower than 2 µg/g DW.

Mauritianin belongs to the group of flavonols. This compound has been well described in the genus *Astragalus* [67,68], but in quinoa, this compound has only been reported in two studies [69,70]. The potential health effects of this compound have not been well described. Mauritianin was confirmed as highly effective against *Candida albicans* [71]. Moreover, an antioxidative effect of mauritianin against DPPH was observed [72]; however, this value was low in comparison to other compounds in the study. The correlation analysis in this study showed that mauritianin is not a very strong contributor to the AA.

Mauritianin had the highest mean content in 2019 (193.86 ± 97.72 µg/g DW). In this year, several extremely high values for this metabolite were observed in the genotypes ‘Cohamamba B’ (540.27 ± 52.78 µg/g DW), and ‘QQ87’ (404.49 ± 11.68 µg/g DW); however, these extremes were not detected in any other year (Figure 4). In contrast, the lowest mean concentration of this compound was detected in 2020 (100.76 ± 43.42 µg/g DW) (Figure 2). The results of mauritianin content reported by Gomez-Caravaca et al. [70] are similar to those measured in the year 2020 in this study. The specific role of mauritianin in plants is not known; nevertheless, the results suggest that the weather conditions in 2019 induced the synthesis of this compound. The genotype ‘Cohamamba B’ had an exceptionally high mauritianin content in all years, apart from 2020, where data were not obtained (Figure 5).

Another abundant flavonol detected in this study was isoquercetin (also referred to isoquercitrin or quercetin 3-glucoside). The highest mean content of isoquercetin was measured in 2018, with 9.10 ± 10.23 µg/g DW (Figure 2). This year also showed notably high values in a total of five genotypes. The genotype ‘QQ056’ had the best performance in this trait, attaining the highest mean isoquercetin content regarding all four years of analysis. The lowest mean isoquercetin content was measured in the year 2021 (2.93 ± 2.44 µg/g DW). In comparison to the available literature, the values measured in this study were considerably higher [20,56].

In contrast to mauritianin, the isoquercetin values showed a relatively low fluctuation throughout the analyzed years between the majority of the genotypes. This suggest that isoquercetin in quinoa is less dependent on the growing conditions in a given year. Nonetheless, geographical variability in the content of this compound was reported in *Cornus* species [73] and *Ceratonia siliqua* L. [74].

Rutin (quercetin-3-rutinoside) was the next most abundant flavonol detected in this study. In quinoa, it was observed to improve plant salinity tolerance through K+ and Na+ regulation in leaf mesophyll [75].

The content of rutin ranged between 0.88 ± 0.03 µg/g DW (genotype ‘Isluga A’) and 19.07 ± 0.61 µg/g DW (genotype ‘QQ056’), both measured in the year 2018. The lowest mean rutin content was measured in 2021 (5.40 ± 3.18 µg/g DW), but a very similar value was also measured in 2020. Accumulation of rutin is impacted by environmental conditions, especially by drought; however, this mechanism has been described in other species but not in quinoa [76,77,78]. In this case, the highest rutin content was observed in 2019 (8.21 ± 3.50 µg/g DW) and the lowest in 2021 (5.40 ± 3.18 µg/g DW). Unlike the results from Pellegrini et al. [56], the content of rutin in our quinoa accessions was lower. On the other hand, similar values to this paper were described in the study of Antognoni et al. [63].

The mean content of the flavonol quercetin ranged between 0.31 ± 0.24 µg/g DW in 2021 and 0.878 ± 1.16 µg/g DW in 2018 (Figure 2). An unusually high value occurred in 2018 in genotype ‘Copacabana A’, reaching 6.48 ± 0.21 µg/g DW. This tendency was also recognized in other years, except for 2021, where this genotype had an average content of quercetin. The contents of quercetin determined in the available literature are quite variable, ranging between 5.27 and 14.30 µg/g DW [64,79,80]. In various plant species, the quercetin level increased due to drought [78], salt [81], and lead stress [82]. Several studies carried out on various plant species concluded that higher quercetin accumulation is a response to increased light exposure and UV-B radiation [83,84], which may partially explain the seasonal variations in the quercetin content observed in our study.

Another minor flavonol identified in this study was kaempferol. Only three genotypes, namely ‘Cahuil A’, ‘Cohamamba A’, and ‘QQ74’, showed the presence of kaempferol in three out of four years of analysis. None of the genotypes showed the presence of kaempferol in all four years. Quercetin, together with kaempferol exhibited a content variability between samples with different geographical origin; therefore, they could be considered metabolic markers [59]. The year 2021 was the least favorable for kaempferol accumulation. Similarly to quercetin, kaempferol synthesis is impacted by light exposure and UV-B radiation [83]. Therefore, the abundant rainfall in 2021 probably decreased the amount of sunlight reaching the quinoa accession, causing a low content of kaempferol.

Lastly, quercitrin (also referred as quercetin 3-rhamnoside, orquercetin 3-O-rhamnoside) was identified in this study; however, trace amounts occurred in only 17 genotypes grown in 2020 and in three genotypes grown in 2019. This compound was previously quantified in the study by Jiang et al. [79]; however, in contrast to our results, the authors indicated quercitrin, together with glycitein, as the major polyphenols in quinoa. In our study, no glycitein was found.

The next group of secondary metabolites detected in this study was the phenolic acids. The most abundant compound from this class was *p*-coumaric acid. The highest content was detected in genotypes ‘Cohamamba B’ (9.72 ± 0.37 µg/g DW in 2018), and ‘Cahuil B’ (7.87 ± 0.24 µg/g DW) in 2020. These genotypes, however, did not performed well in other years. Overall, the lowest content of *p*-coumaric acid throughout all four years was found in the genotypes ‘Red Head A’ and ‘Red Head B’. Different values among genotypes were observed [20,64]; therefore, the reported *p*-coumaric content in the available literature does not correspond to the data obtained in this study.

The year with the highest p-coumaric acid value was 2018 (Figure 2), which may suggest that the synthesis of this compound is upregulated by heat and increased exposure to sunlight, similarly to what was reported in *Nicotiana langsdorffii* Weinmann [85] and hard fescue (*Festuca trachyphylla*) [86]). In comparison to other genotypes, ‘Dave 407B’, ‘Apelawa B1’, and ‘Mint Vanilla’ showed a relatively high stability in *p*-coumaric acid content during the studied years.

Salicylic acid was the next metabolite identified in our study. This important phytohormone regulates several metabolic processes, and the production of metabolites thereby protects the plant against multiple abiotic stresses. For example, it serves as a protection against heat [86] or high contents of heavy metals in the soil [82]. In quinoa, salicylic acid improves salinity tolerance [87] and it increases under UV-B exposure in some genotypes [88]. An unusually high concentration of this metabolite was recognized in the genotypes ‘Apelawa A’ (6.82 ± 0.67 µg/g DW) and ‘Dave 407B’ (4.43 ± 0.25 µg/g DW) in 2019 and 2018, respectively.

Caffeic acid was only found in the quinoa accessions in relatively low quantities (0.09 ± 0.00–0.90 ± 0.07 µg/g DW). The highest amount of this compound was measured in the year 2020 (Figure 2). Galieni et al. [77] reported a higher synthesis of caffeic acid under drought stress. In the sum of precipitation, the year 2020 was not the driest; however, April and July of this year had extremely low rainfall, which could have contributed to the higher accumulation of this phenolic acid.

A very low amount of gallic acid was evaluated in all quinoa genotypes. Increased levels of this phenolic acid were observed in 2020, especially in the genotypes ‘Baer D’ and ‘Cohamamba A’. Furthermore, chlorogenic acid, neochlorogenic acid, and 2-OH-cinnamic acid were identified in quinoa accessions; nonetheless, they were not present in all genotypes and/or they were found in trace amounts. In addition, neochlorogenic acid and 2-OH-cinnamic acid had not been identified in quinoa previously.

The group of flavones was primarily represented by isorhamnetin, with only a trace concentration. This compound was previously reported by Stikic et al. [80] with the content of 3.00 µg/g DW in the genotype ‘Puno’, but with none in the genotype ‘Titicaca’. Other minor compounds detected in this study were apigenin, vitexin, isovitexin, and orientin, which were previously found in other studies [20,89]. Furthermore, homoorientin and luteolin were also detected in minor concentrations; however, they were present only in the year 2021. These compounds had not been described in quinoa before. Nonetheless, all the minor compounds were detected only in a few genotypes. Lastly, rhamnetin was not indicated in any of the analyzed genotypes.

The most abundant compound from the flavanols groups was miquelianin, also named quercetin 3-O-glucuronide or quercetin glucuronide. The level of this compound ranged between 0.26 ± 0.02 µg/g DW (genotype ‘Red Head B’ in 2019) and 33.86 ± 1.10 µg/g DW (genotype ‘QQ056’ in 2018). Similar values were reported by Gomez-Carvaca et al. [90]. The year 2018 showed a total of five extremely high values, for the same genotypes as reported for isoquercetin. In addition, 2018 was also the year with the highest mean concentration of miquelianin (Figure 2). The result of the correlation study showed that miquelianin and isoquercetin had a strong positive association (Figure 4).

Furthermore, epicatechin and taxifolin were quantified only in some quinoa genotypes and during some years, with the highest mean content in 2021. Epicatechin had already been identified in quinoa [64]; however, taxifolin was described here for the first time. Catechin was not detected in this research in any genotype, but it was reported by Tang et al. [8]. Naringenin was the only flavanone detected in this study; however, its amount was negligible in comparison to the other compounds. This compound had not been detected in quinoa previously. Furthermore, hesperidin was screened as well, but its presence was not confirmed, as opposed to in Jiang et al. [79].

The only flavan identified in this study was pinocembrin. This compound was highly accumulated during the year 2020, whereas the lowest mean content was reported in 2021. No pinocembrin was found in the year 2018, except for in the genotypes ‘Baer B’ and ‘QQ57A’. Similar values of pinocembrin content were observed by Garcia Parra et al. [20].

The group of methoxybenzenes was represented by N-feruloyloctopamine (NFO). This compound reached the highest mean concentration in 2020 (3.30 ± 4.32 µg/g DW) (Figure 2). This year showed extremely high values in the genotypes ‘Cohamamba A’ and ‘Tallin A’, which were also observed in 2021. This compound had not been detected or quantified in quinoa before. NFO was reported as an accelerator for cell apoptosis [91] and a promising treatment for hepatocellular carcinoma [92]; however, the role of this metabolite in plants has not been fully elucidated. The results of our research showed a relatively low variability in this compound throughout the analyzed years, which suggests that NFO is less affected by environmental conditions; however, further investigation is needed.

4-OH-benzaldehyde (4-hydroxybenzaldehyde) is the representative of the group of hydroxybenzaldehydes. 4-hydroxybenzaldehyde was previously reported to have antifungal, antiobesity, anti-inflammatory, antiangiogenic, and antinociceptive activities [93,94,95]. The concentration of 4-hydroxybenzaldehyde compound ranged between 0.21 ± 0.01 µg/g DW (genotype ‘Kcoito A’ in 2018) and 5.01 ± 0.22 µg/g DW (genotype ‘QQ87’ in 2021).

Emodin was classified as an anthraquinone. This compound possesses antifungal properties against *Candida albicans* [96]. Several studies also reported anticancer activity [97,98]. In this study, the content of this metabolite was very variable between the years and genotypes. In 2018, only five genotypes contained emodin; as opposed to 2021, in which all genotypes contained this metabolite. To our knowledge, emodin and 4-hydroxybenzaldehyde had never been identified or quantified in quinoa previously. The highest synthesis of both compounds was observed in 2021, which suggest the potential role of these metabolites in quinoa protection against high water levels and/or possible fungal diseases; however, this area requires deeper investigation. Furthermore, genistein and umbelliferon were searched for in this study, but no content of these metabolites was found. The presence of genistein in quinoa was reported by Antognoni et al. [63]. In contrast, umbelliferon was not found in quinoa [99].

### 3.7. PCA Analysis

**Figure 6:**
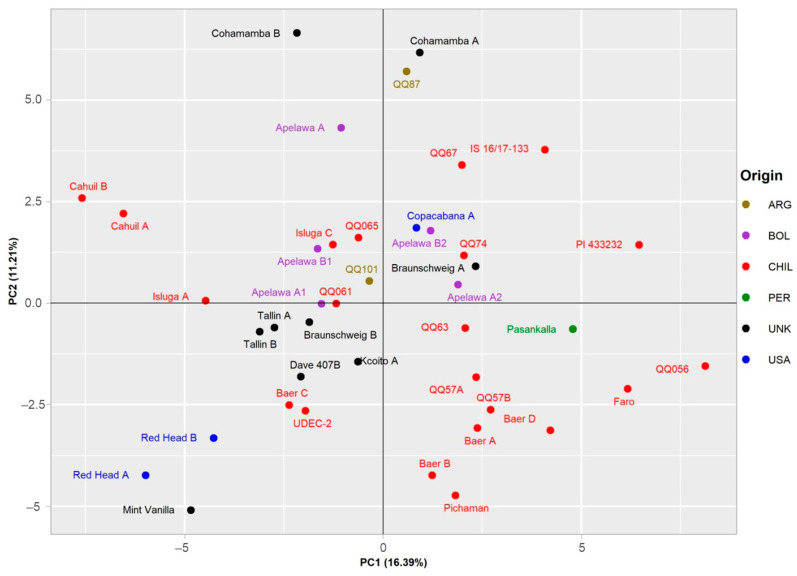
Principal component analysis based on set of 19 descriptors for the set of 41 genotypes. Two main components explaining 16.39% and 11.21% of the total variability, respectively, are displayed. Individual accessions are labeled according to the country of origin, as illustrated in the legend on the right side of the plot.

A PCA representation of the data for the 19 selected descriptors further distinguished between the individual genotypes (Figure 6). In the diagram, the large central group of genotypes of Chilean provenance is located in lower left corner. Of greater interest are the several genotypes located in the outer parts of the plot. The separation of these genotypes suggests the uniqueness of their respective genotypes with respect to the analyzed samples. Although some accessions of the same origin are located close together in the plot, geographical provenance seems to have little to no effect on the spatial distribution of the accessions within the plot. Of the analyzed traits, the separation of genotypes along the first axis, explaining 16.39% of the total variance, is mostly affected by MIQ, TPC, RUT, AA, and IQCE (Supplementary Figure S1). On the other hand, strongest influence on the distribution of genotypes along second axis, explaining 11.21% of the total variance, was from MAU and PCB values.

## 4. Conclusions

For the first time, an extensive collection of 41 quinoa genotypes was evaluated over four years under the environmental conditions of the Czech Republic, Central Europe. The morphological traits of plant height, inflorescence length, and weight of thousand seeds were determined. Most of the quinoa accessions had a better performance in the selected morphological traits in the year 2018, characterized as the driest and with high temperatures in the first half of the growing season.

The crude protein content of quinoa accessions was within the range previously reported for quinoa cultivated in Europe. The protein content was the highest in warm years, but high precipitation significantly affected the protein synthesis. A similar pattern was observed for the accumulation of phenolic compounds. Contrarily, the TPC and AA were enhanced by high rainfall.

A total of 28 metabolites were detected and quantified in quinoa. The most abundant flavonoids were mauritianin, miquelianin, rutin, and isoquercetin. The most abundant contributor to AA was miquelianin. The content of all phenolic compounds varied with the changing weather conditions in the analyzed years, except for isoquercetin, quercetin, and N-feruloyl octopamine, which remained relatively stable values throughout the years of analysis. To our knowledge, six compounds (2-OH-cinnamic acid, homoorientin, luteolin, naringenin, N-feruloyloctopamine, and 4-OH-benzaldehyde) had never previously been identified or quantified in quinoa. A proper selection of appropriate genotypes to accomplish given production aims is needed. Furthermore, the determination of genotype-variable and genotype-stable traits is crucial. Over the four distinct growing periods, the tested genotypes showed a variability in response to different environmental conditions. Nonetheless, the genotypes. ‘Mint Vanilla’, ‘Cahuil A’, ‘Cohamamba B’, and ‘Braunschweig B’ seemed to be less affected by weather conditions in a given year, since they reached high and stable protein contents throughout all four years of analysis in the conditions of the Czech Republic. On the other hand, ‘Braunschweig B’, together with ‘Isluga A’ performed best regarding their stability in weight of a thousand seeds.

Altogether, our results confirm the potential of quinoa as a promising source of nutrients and various bioactive compounds. Furthermore, quinoa is well suited to the climatic conditions of Central Europe. With its ability perform stably or even benefit from periods of hot and drought stress, quinoa might be a potential solution for farmers threatened by the increasing temperatures caused by climatic change.

## Data Availability

Data are contained within the article or the Supplementary Materials.

## Supplementary Materials

The following supporting information can be downloaded at: https://www.mdpi.com/article/10.3390/foods12071440/s1, Table S1. Mass spectrometric data, negative ionization. Table S2. Summarized data of evaluated traits and nutritive compounds (means and standard deviation) of the tested genotypes in all years. Figure S1. The quality of representation of the variables in the factor map. Squared coordinates are displayed. Color scale is proportional to the color key on the right side of the plot.

## Abbreviations

ANOVA—A two-way analysis of variance, AA—Antioxidant activity, C4B—4-hydroxybenzaldehyde, CFA—Caffeic acid, COA—*P*-coumaric acid, DPPH—2,2-diphenyl-1-picrylhydrazyl, ESI—Electrospray ionization, EMO—Emodin, GA—Gallic acid, IL—Inflorescence length, ISR—Isorhamnetin, IQCE—Isoquercetin, KMP—Kaempferol, MAU—Mauritianin, MIQ—Miquelianin, NAR—Naringenin, NFO—N-feruloyloctopamine, PC—Protein content, PCB—Pinocembrin, PH—Plant height, PHE—Sum of phenolic compounds, TE—Trolox equivalent, QCE—Quercetin, RUT—Rutin, SAC—Salicylic acid, TPC—Total phenolic content, WTS—Weight of thousand seeds.
