# Supplementary figures and images for: Nutritional Evaluation of Quinoa Genetic Resources Growing in the Climatic Conditions of Central Europe
Source: Foods. 2023 Mar 28;12(7):1440. doi: 10.3390/foods12071440 (PMC10093933; doi:10.3390/foods12071440)

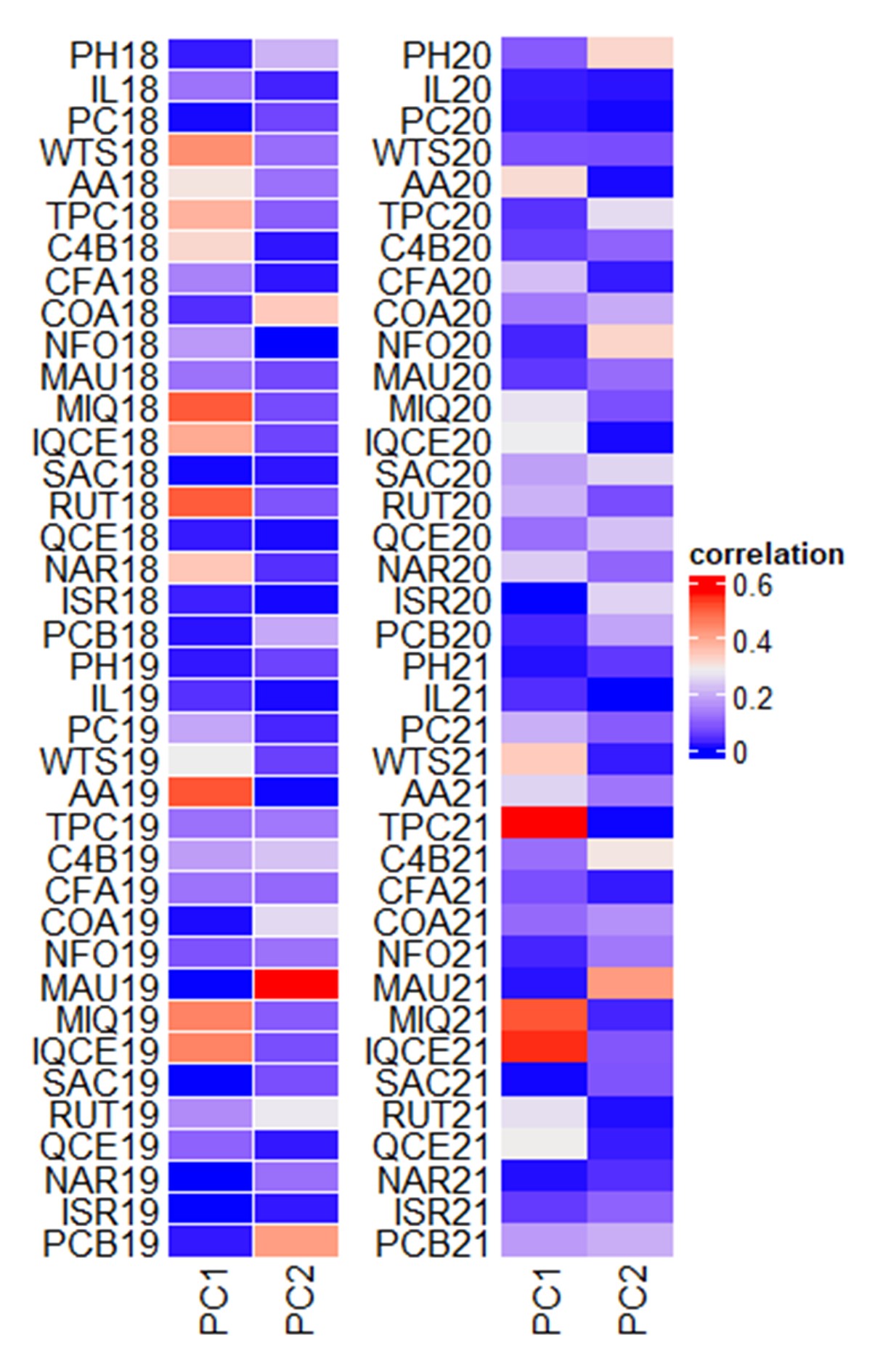

Supplement: Supplementary file 1 [file foods-12-01440-s001.zip › Figure S1.jpg]
